# Dual-Energy CT for Accurate Discrimination of Intraperitoneal Hematoma and Intestinal Structures

**DOI:** 10.3390/diagnostics12102542

**Published:** 2022-10-20

**Authors:** Moritz T. Winkelmann, Florian Hagen, Kerstin Artzner, Malte N. Bongers, Christoph Artzner

**Affiliations:** 1 Department for Diagnostic and Interventional Radiology, University Hospital Tuebingen, 72076 Tuebingen, Germany; 2 Department of Internal Medicine I, Comprehensive Cancer Center, University Hospital Tuebingen, 72076 Tuebingen, Germany

**Keywords:** dual-energy CT, intraabdominal hematomas, virtual unenhanced imaging, iodine maps

## Abstract

The purpose of this study was to evaluate the potential of dual-energy CT (DECT) with virtual unenhanced imaging (VNC) and iodine maps (IM) to differentiate between intraperitoneal hematomas (IH) and bowel structures (BS) compared to linearly blended DECT (DE-LB) images (equivalent to single-energy CT). This retrospective study included the DECT of 30 patients (mean age: 64.5 ± 15.1 years, 19 men) with intraperitoneal hematomas and 30 negative controls. VNC, IM, and DE-LB were calculated. Imaging follow-up and surgical reports were used as references. Three readers assessed diagnostic performance and confidence in distinguishing IH and BS for DE-LB, VNC, and IM. Diagnostic confidence was assessed on a five-point Likert scale. The mean values of VNC, IM, and DE-LB were compared with nonparametric tests. Diagnostic accuracy was assessed by calculating receiver operating characteristics (ROC). The results are reported as medians with interquartile ranges. Subjective image analysis showed higher diagnostic performance (sensitivity: 96.7–100% vs. 88.2–96.7%; specificity: 100% vs. 96.7–100%; *p* < 0.0001; ICC: 0.96–0.99) and confidence (Likert: 5; IRQ [5–5] vs. 4, IRQ [3–4; 4–5]; *p* < 0.0001; ICC: 0.80–0.96) for DECT compared to DE-LB. On objective image analysis, IM values for DECT showed significant differences between IH (3.9 HU; IQR [1.6, 8.0]) and BS (39.5 HU; IQR [29.2, 43.3]; *p* ≤ 0.0001). VNC analysis revealed a significantly higher attenuation of hematomas (50.5 HU; IQR [44.4, 59.4]) than BS (26.6 HU; IQR [22.8, 32.4]; *p* ≤ 0.0001). DE-LB revealed no significant differences between hematomas (60.5 HU, IQR [52.7, 63.9]) and BS (63.9 HU, IQR [58.0, 68.8]; *p* > 0.05). ROC analysis revealed the highest AUC values and sensitivity for IM (AUC = 100%; threshold by Youden-Index ≤ 19 HU) and VNC (0.93; ≥34.1 HU) compared to DE-LB (0.64; ≤63.8; *p* < 0.001). DECT is suitable for accurate discrimination between IH and BS by calculating iodine maps and VNC images.

## 1. Introduction

Hematomas are collections of blood in the extravascular space. Abdominal hematomas can occur anywhere in the abdomen; typical sites include the anterior abdominal wall as rectus sheath hematoma or in the abdominal cavity, e.g., as hemoperitoneum, retroperitoneal, or intramurally in the bowel wall [1,2,3]. In addition, abdominal hematomas may occur due to blunt abdominal trauma, nonaccidental injury, surgery, or spontaneously in anticoagulated patients [4,5].

In addition to initial blood loss with possible circulatory consequences, life-threatening complications can result from intra-abdominal hematomas in the subacute phase and in the long term. Risks arise from the compression of adjacent organs. For this, the intestine and bile ducts prove to be particularly vulnerable. Secondarily, bile ducts could be occluded by compression of the duodenum or biliodigestive anastomosis in postoperative patients [6,7,8]. In addition, intra-abdominal hematomas can become infected or compress venous and portal venous abdominal vessels, leading to thrombosis [9]. As a further complication, intramural hematomas of the intestinal wall may rupture, leading to intestinal perforation and subsequent peritonitis [10]. In the long term, healed intramural hematomas may form scars, which may lead to ileus years later [7]. The treatment of an intraperitoneal hematoma includes surgery and the placement of drains. Drains can be further enhanced by intracavitary thrombolysis with urokinase and recombinant tissue plasminogen activators [11,12].

In recent years, assessing patients with a suspected intra-abdominal hematoma has shifted toward noninvasive methods. Computed tomography (CT) is the standard procedure for determining the presence, extent, and precise location of intra-abdominal hematomas [13]. Unfortunately, the detection on CT images is not trivial, as single energy CT (SECT) numbers of clotted blood (45–70 HU) strongly overlap with small bowel structures’ CT numbers (≈70 HU) [14,15]. Furthermore, hematomas’ attenuation changes with age and the distance from the source of initial bleeding. Therefore, exploratory laparotomy is performed in many cases of diagnostic doubt.

Early and accurate diagnosis of hematomas is critical to avoid delayed initiation of therapeutic measures, such as surgical evacuation, or to avoid additional examinations that could lead to increased radiation exposure, costs, and complication risks.

In contrast to SECT, dual-energy CT (DECT) provides additional information on material-specific attenuation. In particular, iodine contrast can be quantitatively detected, allowing for the calculation of synthetic virtual non-amplified imaging (VNC), as well as calculating iodine maps (IM) [16,17,18,19].

We hypothesized that quantitative DECT analysis would allow reliable discrimination between physiologic bowel structures and hematomas.

This single-center retrospective study aimed to evaluate the potential of DECT with IM and VNC to improve the differentiation between intraperitoneal hematomas and adjacent bowel structures when compared to SECT.

## 2. Materials and Methods

### 2.1. Patient Population

**Figure 1:**
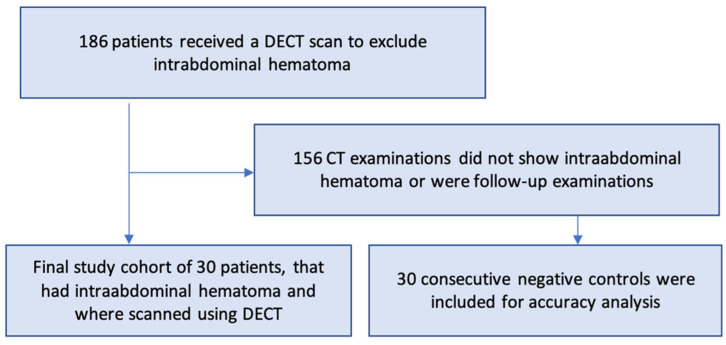
Flowchart of the study cohort selection. Of note, DECT: dual-energy CT.

The local review board of our university hospital approved this HIPAA-compliant retrospective study with a waiver of written informed consent. Our institutional patient database was searched to identify patients with intra-abdominal hematomas who received clinically indicated abdominal DECT between June 2018 and April 2021. Patients with relevant intraperitoneal hematomas near the bowel structures were included. Patients with abdominal wall hematomas and hematomas in major upper abdominal organs, such as the liver and stomach, were excluded. The final cohort included 30 patients with a mean age of 64.5 ± 15.1 years. The causes of intra-abdominal hematomas were complications after previous surgical procedures (*n* = 19), complications of infection/tumor disease (*n* = 5), post-traumatic (*n* = 3), or spontaneous development (*n* = 3) (Table 1). In all patients, regression of the hematoma was monitored by CT follow-up. Laparotomy was required to remove the hematoma in 19 cases, whereas interventional treatment (e.g., CT drainage) was performed in 5 patients. To perform an accuracy analysis, 30 consecutive patients without an intra-abdominal hematoma were included; please see Figure 1.

### 2.2. Reference Standard

The clinical reference standard was established by a radiologist with ten years of experience in abdominal CT imaging. The reader had access to all clinical information systems and histopathological findings after surgical removal of the hematomas. The reference standard was based on surgical reports of hematoma removal (*n* = 19), subsequent interventional treatment (e.g., CT drainage; *n* = 5), and subsequent CT scans (*n* = 30).

Research manuscripts reporting large datasets that are deposited in a publicly available database should specify where the data have been deposited and provide relevant accession numbers. If the accession numbers have not yet been obtained at the time of submission, please state that they will be provided during the review. They must be provided prior to publication.

### 2.3. Acquisition Parameters

DECT was performed on a third-generation dual-source CT system (SOMATOM Force; Siemens Healthcare, Erlangen, Germany). All images were acquired in the portal venous phase 80 s after administration of a body weight-adapted contrast media (0.5 mL per kilogram body weight, Imeron 400 mg iodine/mL; Bracco, Milan, Italy) at a flow rate of 2.0 ± 0.5 mL/s using a dual-syringe injector (Medrad; Bayer), followed by a saline chaser (40 mL). Additional unenhanced CT and arterial phase imaging were performed only when active bleeding was suspected. Automatic current modulation was used (CareDose4D; Siemens Healthineers). The DECT settings were 100 kVp for tube A and tin-filtered (Sn) 150 kVp for tube B, with a reference tube current time product of 190 mAs and 95 mAs, respectively. The collimation was 0.6 × 192 mm with a pitch factor of 0.6 and a gantry rotation time of 0.5 s. All DECT series were reconstructed on a DECT workstation (Syngo.Via VB10B, Siemens Healthineers) as axial and coronal slices with 1.5-mm slice collimation. Images were analyzed using an image analysis workstation (Syngo.Via, version VB10B, Siemens Healthcare, Erlangen, Germany). An iodine subtraction algorithm (Liver VNC in Syngo.Via, version VB10B, Siemens Healthcare, Erlangen, Germany) was used to calculate IM, VNC, and CT-LB (equal to SECT) images. For this purpose, the software performs three-material decomposition using spectral analysis of image data from the high- and low-energy X-ray spectrum.

### 2.4. Subjective Image Analysis

All subjective readings took place at clinical workstations equipped with diagnostic monitors. Subjective image analysis was performed by three readers with five (reader 1), three (reader 2), and ten years (reader 3) of abdominal imaging experience. The results of the subjective image analysis were documented using Excel spreadsheets. The readers were allowed to scroll and window freely through the CT datasets to provide the most authentic reading possible.

#### 2.4.1. Diagnostic Performance

All readers assessed the entire study cohort consisting of 30 patients with intra-abdominal hematomas with spatial association to intestinal loops and 30 negative controls for the presence of intra-abdominal hematomas. The cases were presented in random order, and the readers were blinded to the final diagnoses. This reading was done in the first step based on DE-LB (equal to SECT). After a washout period of 3 months, a second reading was performed based on IM in a newly randomized fashion.

#### 2.4.2. Diagnostic Confidence

After another washout period of 3 months, the three readers assessed how well they could discriminate between intraperitoneal hematomas and physiologic bowel structures in all 30 true positive cases with DE-LB. To avoid recall bias, the cases were previously re-randomized. After another 3-month washout period, the three readers assessed the same question using the IM. Diagnostic confidence in distinguishing bowel structures from extraluminal hematomas was graded using a five-point Likert scale (1: very poor, 2: poor, 3: acceptable, 4: good, and 5: very good).

### 2.5. Objective DECT Image Analysis

True positive images were analyzed using an image analysis workstation (Syngo.Via, version VB10B, Siemens Healthineers, Erlangen, Germany). An iodine subtraction algorithm (Liver VNC in Syngo.Via, version VB10B, Siemens Healthineers, Erlangen, Germany) was used to perform a three-material decomposition using spectral analysis of the image data of the high- and low-energy X-ray spectrum. As a result, the attenuation in each voxel can be decomposed into the components fat, soft tissue, and iodine [20].

**Figure 2:**
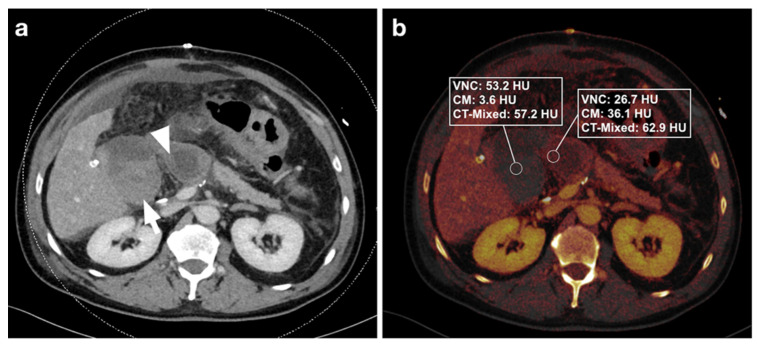
(**a**) 59-year-old male patient with intra-abdominal hematoma (arrow) adjacent to small bowel loops (arrowhead) after Whipple surgery for pancreatic cancer. (**b**) Measurements in iodine maps show a difference in VNC and iodine values (dual-energy CT) between hematoma and intestinal loops, but no difference in DE-LB (single-energy CT). After CT, the patient was reoperated, and the hematoma was removed.

**Figure 3:**
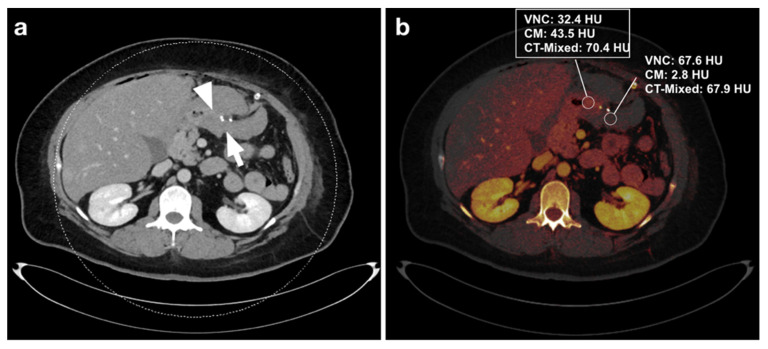
(**a**) 46-year-old male patient with intra-abdominal hematoma (arrow) adjacent to small bowel loops (arrowhead) after gastric tube surgery. (**b**) Measurement of iodine maps shows a difference in VNC and iodine values (dual-energy CT) between hematoma and intestinal loops, but no difference in DE-LB (single-energy CT). This case was incorrectly rated as negative in the DE-LB procedure by all 3 readers. After CT, the patient underwent laparoscopy with hematoma evacuation.

**Figure 4:**
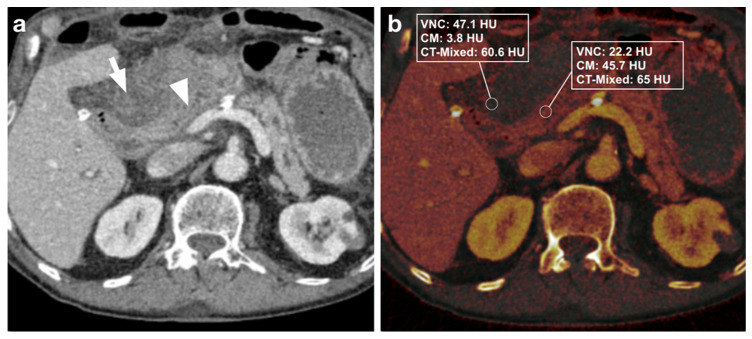
(**a**) 81-year-old male patient with intra-abdominal hematoma (arrow) adjacent to a small bowel loop (arrowhead) after Whipple surgery. (**b**) Measurement of iodine maps shows a difference in VNC and iodine values (dual-energy CT) between hematoma and intestinal loops, but no difference in DE-LB (single-energy CT). Due to the extensive hematoma, a re-laparotomy was subsequently performed.

**Figure 5:**
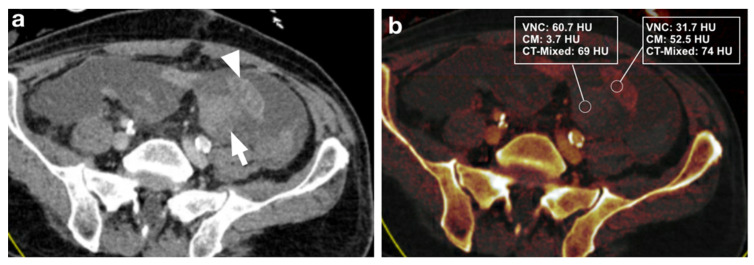
63-year-old male patient after a two-stage colectomy following mesenteric infarction with intestinal ischemia. (**a**) Intra-abdominal hematoma (arrow) adjacent to a small bowel loop (arrowhead). (**b**) Measurement of iodine maps shows a difference in VNC and iodine values (dual-energy CT) between hematoma and intestinal loops, but no difference in DE-LB.

Three distinct regions of interest (ROIs) were placed in the bowel wall and the adjacent intraperitoneal hematoma. DECT-derived VNC, IM, and DE-LB values were documented (Figure 2, Figure 3, Figure 4 and Figure 5). Measurements were performed by a reader with ten years of abdominal CT imaging experience. The readers had access to the clinical reference standard for the correct identification of hematoma and intestinal loops.

### 2.6. Statistics

Statistical analysis of mean values from DECT image analysis was performed using JMP 14 (SAS Institute Inc., Cary, NC, USA), MedCalc Statistical Software 18.1 (MedCalc Software bvba, Ostend, Belgium), and SPSS (SPSS Statistics 26, IBM Corp., Armonk, NY, USA).

Because of the nonnormal distribution of the data, nonparametric tests (Wilcoxon test) were performed to compare the means of IM, VNC, and CTLB images of hematomas and physiologic bowel structures. The Wilcoxon paired-samples test was used to calculate the differences in diagnostic confidence between the DE-LB and DECT methods. To compare the diagnostic performance of DE-LB and DECT, cross-tabulations with 95% confidence intervals were evaluated to calculate sensitivity, specificity, NPV, and PPV. Interobserver agreement on subjective image analysis was determined by interclass correlation coefficients (ICCs) and 95% CIs based on a single-rating, absolute-agreement, two-way random-effects model. ICC values below 0.5 indicate poor reliability, values between 0.5 and 0.75 indicate moderate reliability, values between 0.75 and 0.9 indicate good reliability, and values above 0.90 indicate excellent reliability. Receiver operating characteristics (ROC) were used to evaluate the diagnostic performance of the DECT image analysis. The results are reported as medians with interquartile ranges.

## 3. Results

### 3.1. Subjective Image Analysis

#### 3.1.1. Diagnostic Performance

For all three readers, DECT-based IM (sensitivity: 96.7–100%, specificity: 100%) had higher diagnostic accuracy than DE-LB (sensitivity: 90.9–96.7%, specificity: 96.7–100%). Reader 1 missed the intra-abdominal hematoma in three cases with DE-LB and had no false-positive cases, whereas no hematoma was missed with IM. In DE-LB mode, Reader 2 missed an intraperitoneal hematoma in four cases and scored one case as a false positive. In contrast, in IM, only one hematoma was missed, and no case was rated as a false positive. Reader 3 classified one case as a false negative in the DE-LB mode and had no false positives, whereas no hematoma was missed in the IM (Table 2). One case was falsely graded as negative in DE-LB mode by all 3 readers (Figure 3a,b). ICC for diagnostic performance was excellent with values ranging from 0.96 to 0.99 (*p* < 0.001).

#### 3.1.2. Diagnostic Confidence

When comparing diagnostic confidence in distinguishing intra-abdominal hematomas from physiologic bowel structures, DECT performed significantly better than DE-LB (*p* < 0.0001). All three readers achieved a mean Likert score of 5 (interquartile range [IQR]: 5–5) when using IM images compared to a Likert score of 4 (IQR 3–4 for reader 1; IQR 4–5 for readers 2 and 3) when using the DE-LB images (*p* < 0.0001). ICC for diagnostic confidence scores were good to excellent, with values ranging from 0.80 to 0.96 (*p* < 0.001).

### 3.2. Objective Image Analysis

#### 3.2.1. DECT Image Analysis

**Figure 6:**
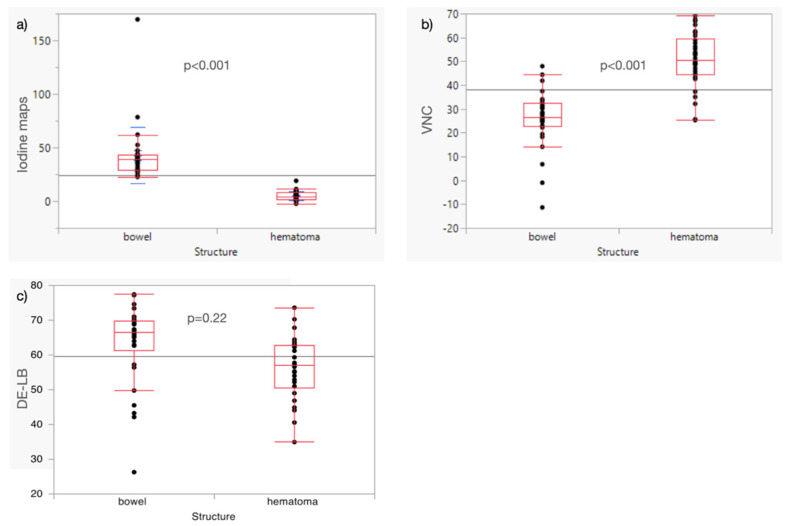
Boxplots show median and quantiles of IM (**a**), VNC (**b**), and DE-LB (**c**). Of note: IM: dual-energy CT-based iodine maps; VNC: dual-energy CT-based virtual non-contrast images; DE-LB: linearly blended dual energy CT images equal to single energy CT images.

Analysis of iodine maps revealed significant differences between physiologic bowel structures (39.5 HU; IQR [29.2, 43.3]) and intraperitoneal hematomas (3.9 HU; IQR [1.6, 8.0]; *p* < 0.001). VNC showed significant differences between physiological bowel structures (26.6 HU; IQR [22.8, 32.4]) and intraperitoneal hematomas (50.5 HU; IQR [44.4, 59.4]; *p* < 0.001). DE-LB showed no significant differences between hematomas (60.5 HU; IQR [52.7, 63.9]) and intestinal structures (63.9 HU; IQR [58.0, 68.8]; *p* = 0.22; Figure 6a–c).

#### 3.2.2. Diagnostic Performance

**Figure 7:**
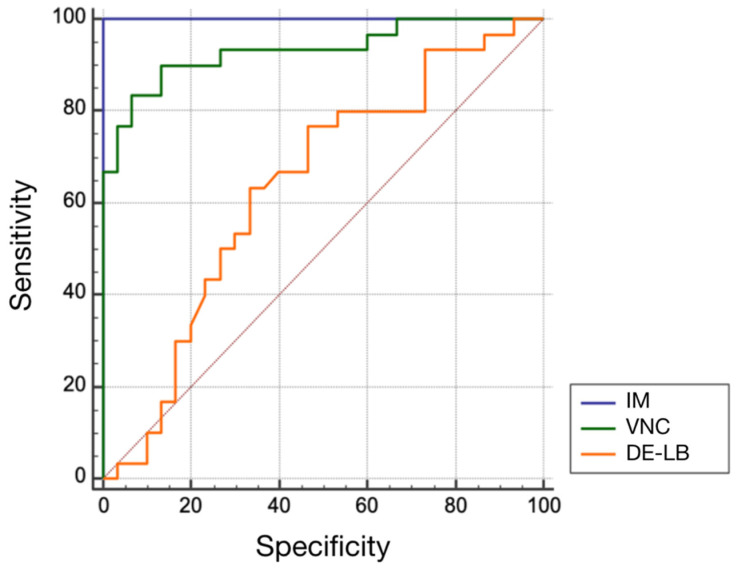
Comparison of ROC curves for IM, VNC, and DE-LB.

ROC analysis for the diagnostic potential in distinguishing intraperitoneal hematomas from physiologic bowel structures revealed significantly higher AUC values and increased sensitivity for IM (AUC = 1.0; sensitivity = 100%; specificity = 100%; optimal threshold by Youden index < 19 HU) and VNC (AUC = 0.93; sensitivity = 90%; specificity = 86.7%; optimal threshold > 34.1 HU) (both *p* < 0.001) compared to DE-LB (AUC = 0.64; sensitivity = 76.7%; specificity = 53.3%; optimal threshold < 63.8 HU) (Figure 7).

## 4. Discussion

The purpose of this study was to evaluate the diagnostic accuracy of DECT for distinguishing subacute intraperitoneal hematomas from physiologic bowel structures by extracting DECT-based IM and VNC. DECT is a robust modern technology that has been the subject of several studies to determine tissue composition based on differential attenuation due to Compton and photoelectric effects caused by the variable attenuation of high- and low-energy X-ray spectra [21,22]. Based on our data, we were able to increase the mean of 3 readers’ sensitivity for correct diagnosis of intraabdominal hematoma from 93.9% to 98.9%. At the same time, the mean specificity also developed positively, from 98.9% to 100%, with excellent agreement from all three readers (ICC 0.96–0.99). Since no comparable studies are available for this question, the correctness of our findings can at best be substantiated by an increase in sensitivity and specificity in the diagnosis of intracerebral hemorrhages in the field of neuroradiology [23,24]. Otherwise, the advantages of dual-energy CT in intestinal bleeding or post-traumatic hemorrhage detection were only published as case illustrations without systematically examining the diagnostic performance [25,26]. Although we were able to significantly increase sensitivity by using DECT, it was already high with DE-LB. This is important because single-energy CT remains a widely used, rapid, and robust method for detecting and characterizing intra-abdominal hematomas, especially in institutions where DECT is not available. Furthermore, we defined exact threshold values based on ROI analysis, which enabled a reliable distinction between hematoma and intestinal loop. The evaluation of the IM and VNC images was remarkably accurate. Here, the area under the curve of the ROC analysis for IM (AUC = 1.00) and VNC (AUC = 0.93) could be significantly increased compared to the pure density-based evaluation of the DE-LB images (equal to single-energy CT, AUC = 0.64). It is particularly encouraging that we were able to define thresholds based on the Youden index, for which we obtained a sensitivity of 100% and a specificity of 100% of IM (optimal threshold < 19 HU) and a sensitivity of 90.0%, and a specificity of 86.7% of VNC (optimal threshold of >34.1 HU). This significantly contrasts with a sensitivity of 76.7% and a specificity of 53.3% of CT-LB images (optimal threshold < 63.8 HU).

Another positive aspect of this diagnostic approach is the possible saving of X-rays by omitting a truly native CT acquisition, which has already been worked out in many preliminary studies. This has been shown in the context of a hemorrhage search or organ lesion characterization [22,27]. DECT-based IM can be considered surrogate parameters for one-shot tissue perfusion and has shown promising results in several studies, including the diagnosis of early acute pancreatitis and lymph node metastases [19,28]. Although intra-abdominal hematomas, e.g., after abdominal surgery, are relatively rare and depend on the type of procedure, accurate diagnosis is crucial because, as described above, serious complications can occur in the clinical course. Contrast-enhanced DECT quantification of IM and VNC can help to accurately differentiate intra-abdominal hematomas from intestinal structures in a single portal venous phase of abdominal CT and to determine their precise extent. This may be of particular clinical relevance when a CT scan was performed exclusively in the portal venous phase in the presence of nonspecific abdominal symptoms or when only a rapid split-bolus protocol was examined in the context of an emergency room diagnosis without acquiring additional native images. In this case, dual-energy post-processing can avoid time-consuming additional examinations. In addition to the reduction in radiation exposure, as mentioned above, an accurate diagnosis can be made more quickly. Other studies have shown that DECT, with its post-processing applications, such as iodine-selective imaging and virtual monoenergetic imaging, can reliably demonstrate the detectability of traumatic and nontraumatic visceral and vascular emergencies on abdominal CT [26,29]. To our knowledge, this study is the first to investigate and demonstrate the diagnostic performance of DECT in distinguishing intra-abdominal hematomas and bowel structures, which is challenging for the reading radiologist, especially in emergency cases. Therefore, this study’s results may help increase diagnostic accuracy in clinical practice and avoid severe complications with a significantly prolonged length of clinical stay.

We are aware that our study has limitations. A retrospective study design was used, and the study cohort was small. Because of potential case selection bias, diagnostic performance in this retrospective cohort should be viewed with caution. Nevertheless, the results of this study should help determine the extent of intra-abdominal hematoma and facilitate the problematic delineation of adjacent bowel structures on abdominal CT in the portal venous phase, especially in patients with acute abdomens. Patients in this study were not suspected of active bleeding before the CT examination, so the results presented should not be interpreted in this context. In addition, quantitative analysis of IM and VNC may not be a substitute for other methods for the definitive diagnosis of intra-abdominal hematomas, such as diagnostic laparoscopy.

In conclusion, IM and VNC of single-phase abdominal dual-energy CT can accurately and confidently differentiate intraabdominal hematomas from bowel structures compared with single-energy CT.

## Figures and Tables

**Table 1:** Patient characteristics.

Variables	N	%	N	%
Study cohort			Negative control group	
No. of patients	30		30 *	
Age, years, mean ± SD	64.5 ± 15.1 years		62.3 ± 13.2 years *	
Gender				
Male	19	63	18	60 *
Female	11	37	12	40 *
Causes of intra-abdominal hematoma				
Post-surgical	19	63		
Infection/tumor disease	5	17		
Traumatic Spontaneous development	3 3	10 10		

Of note: * *p* > 0.05; SD standard deviation.

**Table 2:** Diagnostic performance of DECT vs. DE-LB to diagnose intraabdominal hematomas.

		Sensitivity (95% CI)	Specificity (95% CI)	PPV (95% CI)	NPV (95% CI)
Reader 1	DE-LB	90.9% (74.5–97.6)	100% (85.9–100)	100% (0.85–1)	90.1 (74.5–97.6)
	IM	100% (85.9–100)	100% (85.9–100)	100% (85.9–100)	100% (85.9–100)
Reader 2	DE-LB	88.2% (71.6–96.2)	96.7% (82.5–99.8)	96.7% (0.82–0.99)	88.2% (71.6–96.2)
	IM	96.7 (81.5–99.8)	100% (85.9–100)	100% (85.9–100)	96.7 (81.5–99.8)
Reader 3	DE-LB	96.7 (81.5–99.8)	100% (85.9–100)	100% (85.9–100)	96.7 (81.5–99.8)
	IM	100% (85.9–100)	100% (85.9–100)	100% (85.9–100)	100 (85.9–100)

Of note: PPV = positive predictive value; NPV = negative predictive value; DE-LB linearly blended dual-energy CT images equal to single-energy CT; IM: dual energy CT-based iodine maps.

## Data Availability

Data sharing not applicable.
